# The Effect of Hyperbaric Oxygen Therapy on Markers of Oxidative Stress and the Immune Response in Healthy Volunteers

**DOI:** 10.3389/fphys.2022.826163

**Published:** 2022-01-31

**Authors:** Silke D. de Wolde, Rick H. Hulskes, Stijn W. de Jonge, Markus W. Hollmann, Robert A. van Hulst, Robert P. Weenink, Matthijs Kox

**Affiliations:** ^1^Department of Anesthesiology, Amsterdam UMC, University of Amsterdam, Amsterdam, Netherlands; ^2^Department of Hyperbaric Medicine, Amsterdam UMC, Location AMC, Amsterdam, Netherlands; ^3^Department of Surgery, Amsterdam UMC, Location AMC, Amsterdam, Netherlands; ^4^Department of Intensive Care Medicine, Radboud University Medical Center, Nijmegen, Netherlands; ^5^Radboud Center for Infectious Diseases (RCI), Radboud University Medical Center, Nijmegen, Netherlands

**Keywords:** hyperbaric oxygenation, hyperbaric oxygen therapy, immune response, inflammation, oxidative stress, immune system, cytokines, healthy volunteers

## Abstract

Hyperbaric oxygen therapy (HBOT) consists of breathing 100% oxygen under increased ambient pressure. There are indications that HBOT induces oxidative stress and activates immune pathways. However, previous research on immunological effects of HBOT has mainly been established in *in vitro* experiments and selected patient populations, limiting generalizability and increasing the chances of confounding by comorbidities and specific patient-related factors. More insight into the immunological effects of HBOT would aid investigation and comprehension of potentially novel treatment applications. Therefore, in this study, we investigated the effects of three 110-min HBOT-sessions with 24-h intervals on immunological parameters in healthy, young, male volunteers. Blood samples were obtained before and after the first and third HBOT sessions. We assessed neutrophilic reactive oxygen species (ROS) production, systemic oxidative stress [plasma malondialdehyde (MDA) concentrations] as well as neutrophil phagocytic activity, plasma concentrations of tumor necrosis factor (TNF), interleukin (IL)-6, IL-8, and IL-10, and production of TNF, IL-6, and IL-10 by leukocytes *ex vivo* stimulated with the Toll-like receptor (TLR) ligands lipopolysaccharide (TLR4) and Pam3Cys (TLR2). We observed decreased neutrophilic ROS production and phagocytosis following the second HBOT session, which persisted after the third session, but no alterations in MDA concentrations. Furthermore, plasma concentrations of the investigated cytokines were unaltered at all-time points, and *ex vivo* cytokine production was largely unaltered over time as well. These results indicate no induction of systemic oxidative stress or a systemic inflammatory response after repeated HBOT in healthy volunteers but may suggest exhaustion of ROS generation capacity and phagocytosis.

## Introduction

Hyperbaric oxygen therapy (HBOT) consists of breathing 100% oxygen under increased ambient pressure (Moon, 2019). The resulting systemic hyperoxia leads to, among other things, an antimicrobial effect, improved neovascularization, improved post-ischemic tissue survival, and vasoconstriction (Camporesi and Bosco, 2014; Shinomiya, 2019; Ürütük, 2020). These beneficial effects of HBOT are used to treat several diseases, including carbon monoxide poisoning, decompression illness, gas embolism, and necrotizing soft tissue infections (Mathieu et al., 2017; Moon, 2019). In addition, some clinical studies have suggested a positive role of hyperbaric oxygen preconditioning in reducing infections and shortening hospital stay after surgery (Yogaratnam et al., 2010; Li et al., 2011; Bosco et al., 2014; Friedman et al., 2019).

Many of the currently used and potential new indications for HBOT rely on its putative effects on the immune system, including the generation of reactive oxygen species (ROS), which constitutes an important antimicrobial defense mechanism (Camporesi and Bosco, 2014; Shinomiya, 2019; Ürütük, 2020). This effect on the immune system may be especially relevant for the possible use of HBOT in preconditioning before surgery. Most studies on the interaction between HBOT and the inflammatory pathway have been performed *in vitro*, in animals, or specific patient populations. For instance, *in vitro* exposure of a neutrophil-like cell line to HBOT was shown to increase phagocytic activity (Almzaiel et al., 2013). In patients with unilateral femoral head necrosis, an increase in plasma ROS production was found following a large number of HBOT sessions and a gradual decrease in plasma levels of tumor necrosis factor (TNF) and interleukin (IL)-6 was observed (Bosco et al., 2018). Likewise, a decrease in TNF plasma levels was observed following HBOT in CO-poisoned patients (Vezzani et al., 2016). Little research has been performed concerning the immunological effects of HBOT in healthy volunteers, although this may yield more clear-cut insights due to the lack of comorbidities or specific patient-related factors.

In the present study, we evaluated the effects of repeated HBOT on immunological parameters in healthy, young, male volunteers to gain more insight into immunological effects of HBOT to facilitate potential novel treatment applications.

## Materials and Methods

This study was conducted according to the principles of the declaration of Helsinki and in accordance with the Medical Research Involving Humans Subjects Act. Approval was given by the Medical Research Ethics Committee of our institution (NL69684.018.19). All subjects provided written informed consent.

### Subjects

In total, 11 volunteers were recruited for participation in this study via adverts in the Amsterdam University Medical Centers (Amsterdam UMC), location AMC. Participants met the following inclusion criteria: (1) male and (2) aged 18–40 years. Subjects who were (1) not proficient in the Dutch language, (2) unable to give informed consent, or (3) unfit for HBOT as assessed by a hyperbaric physician were excluded from this study.

### Study Protocol and Blood Sampling

Three HBOT-sessions with 24-h intervals were performed. Each session had a duration of 110 min, consisting of a 15-min pressure build-up, 75 min of breathing 100% oxygen through a tight-fitting facemask at a pressure of 240 kPa (2.4 bar) with two 5-min breaks of breathing normal air, and a 10-min pressure reduction. Venous blood samples were obtained using ethylenediaminetetraacetic acid (EDTA) and lithium heparin (LH) tubes (Vacutainer System; BD Biosciences, Plymouth, United Kingdom) directly before session one (T1), directly after session one (T2), directly before session three (T3) and directly after session three (T4).

### Neutrophilic Reactive Oxygen Species Generation Assay

To quantify neutrophilic ROS production, we performed a dihydrorhodamine (DHR)-based flow cytometric assay. One-hundred μL LH-anticoagulated whole blood was incubated with 500 ng/mL DHR (Sigma-Aldrich, Saint Louis, MO, United States) in the absence and presence of phorbol 12-myristate 13-acetate (PMA, 20 ng/mL, Sigma-Aldrich, to elicit a maximum respiratory burst) for 15 min at 37^°^C, with gentle mixing every 5 min. Subsequently, erythrocytes were lysed using an NH_4_Cl solution, samples were washed, and neutrophils were stained with a CD16-PC7 antibody (Biolegend, San Diego, CA, United States). After washing, samples were measured on a BD Biosciences FACSCanto II flow cytometer. Data were analyzed using Kaluza software (Beckman Coulter, Fullerton, CA, United States). Neutrophils were gated using PC7 fluorescence, and ROS production was quantified by determining the MFI of the gated neutrophils in the FITC channel.

### Malondialdehyde Plasma Levels

Plasma MDA levels were determined to measure lipid peroxidation of plasma proteins, reflecting systemic oxidative stress. Immediately after withdrawal, EDTA-anticoagulated blood was centrifuged (2,000 g for 10 min at 4°C), after which plasma was stored at –80°C until analysis using the Thiobarbituric Acid Reactive Substances (TBARS) Parameter Assay Kit (R&D Systems, Abingdon, United Kingdom) following the manufacturer’s instructions.

### Neutrophil Phagocytosis Assay

Neutrophil phagocytosis was measured using the pHrodo Red *E. coli* and pHrodo Green *S. aureus* BioParticles Phagocytosis Kits for Flow Cytometry according to the manufacturer’s instructions (Life Technologies, Bleiswijk, Netherlands). Briefly, 100 μL LH-anticoagulated blood was incubated with pHrodo Red *E. coli* and pHrodo Green *S. aureus* BioParticles for 20 min at 37^°^C. Thereafter, erythrocytes were lysed using the buffer supplied with the kit. Subsequently, samples were washed, and neutrophils were stained with a CD16-PC7 antibody (Biolegend, San Diego, CA, United States). After washing, samples were measured on a BD Biosciences FACSCanto II flow cytometer. Data were analyzed using Kaluza software (Beckman Coulter, Fullerton, CA, United States). Neutrophils were gated using PC7 fluorescence, and phagocytosis was quantified by determining the MFI of the gated neutrophils in the FITC (pHrodo Green *S. aureus*) and PE (pHrodo Red *E. coli*) channels.

### Plasma Cytokine Concentrations

Immediately after withdrawal, EDTA-anticoagulated blood was centrifuged (2,000 g for 10 min at 4°C), after which plasma was stored at –80°C until analysis. Concentrations of the cytokines, TNF, IL-6, IL-8, and IL-10 were analyzed batchwise (i.e., all samples were measured in one batch using a single assay on the same day to preclude inter-assay variability) using a Luminex assay (detection range: 3.2–10.000 pg/ml) following the manufacturer’s instructions (Milliplex, Millipore, Billerica, MA, United States).

### *Ex vivo* Whole Blood Stimulation

Leukocyte cytokine production capacity was assessed by *ex vivo* whole blood stimulation with LPS (TLR4 ligand) and Pam3Cys (TLR2 ligand) using an in-house developed system (Kox et al., 2012). Briefly, 0.5 mL LH-anticoagulated blood was added to pre-filled tubes with 2 mL culture medium or 2 mL culture medium supplemented with 12.5 ng/mL LPS (final concentration after addition of blood: 10 ng/mL) or 1.25 μg/mL Pam3Cys (final concentration after addition of blood: 1 μg/mL). After 24 h of incubation at 37°C, samples were centrifuged, and supernatants were stored at −80°C until batchwise analysis. Concentrations of TNF, IL-6, and IL-10 were determined using ELISA according to the manufacturer’s instructions (Duoset, R&D systems, Minneapolis, MN, United States).

### Statistical Analyses

All measured parameters were tested for normality using the Shapiro-Wilk test. Demographic data are expressed as the mean ± standard error of the mean (SEM), and the investigated immunological parameters are represented as geometric mean with a 95% confidence interval (CI). For analysis of differences between time points, mixed model analyses were used after log-transformation, corrected with the method of Greenhouse and Geisser. This mixed model used a compound symmetry covariance matrix and was fitted using Restricted Maximum Likelihood (REML). *Post hoc* tests were corrected for multiple comparisons by controlling the False Discovery Rate. Corrected *p*-values < 0.05 were considered significant. Statistical analyses were performed using GraphPad Prism 9.1 (GraphPad Software, San Diego, CA, United States).

## Results

### Study Population

Of the 11 eligible volunteers, 10 were included in the final analysis. One subject reported flu-like complaints during and after the HBOT sessions, likely induced by a recent influenza immunization. Because of this external immunomodulating event, we decided to exclude this participant from the final analysis. HBOT was well-tolerated by all other volunteers, and no adverse events were recorded during or after the study. The age of the study group was 22.90 ± 0.71 years, and the mean body mass index (BMI) was 24.22 ± 0.47.

### Neutrophilic Reactive Oxygen Species Generation

**FIGURE 1:**
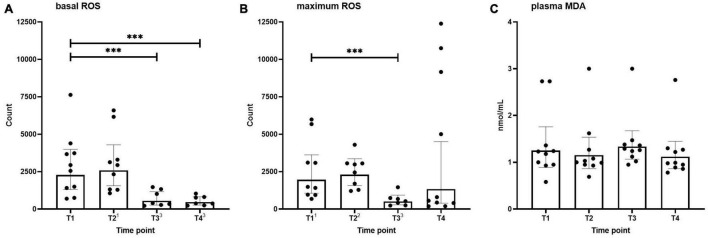
Basal **(A)** and maximum **(B)** neutrophilic ROS generation, and MDA levels **(C)** in healthy volunteers measured before hyperbaric oxygen therapy (HBOT) session one (T1), after HBOT-session one (T2), before HBOT-session three (T3), and after HBOT-session three (T4). Data are represented as scatter plots with bars indicating geometric mean and whiskers representing 95% confidence interval (CI). Superscript font represents the number of missing values due to technical issues at each time point. ^***^p < 0.001 vs. T1.

Basal and maximum (PMA-stimulated) neutrophilic ROS generation across the different time points are shown in Figure 1. Basal ROS generation was not altered after the first HBOT session (T2) but was significantly decreased before and after the third session (T3 and T4, Figure 1A). A similar pattern was observed for the maximum respiratory burst induced by PMA, although this did not reach statistical significance for the T4 time point (Figure 1B).

### Systemic Oxidative Stress

Plasma MDA levels as an indicator of systemic oxidative stress across the different time points are shown in Figure 1C. No differences were found over time in MDA concentrations.

### Neutrophil Phagocytosis

**FIGURE 2:**
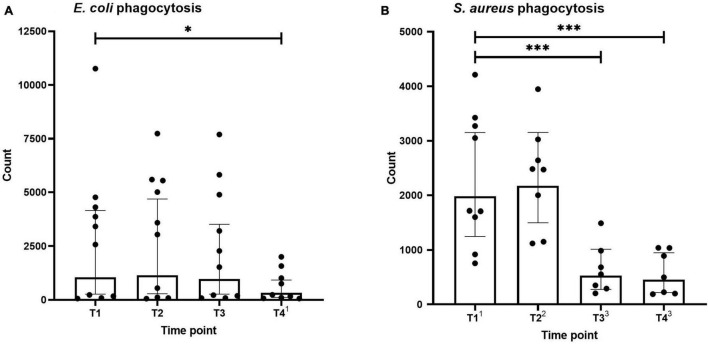
Phagocytosis of *Escherichia coli* **(A)** and *Staphylococcus aureus* **(B)** by neutrophils. Data are represented as scatter plots with bars indicating geometric mean and whiskers representing 95% confidence interval (CI). Superscript font represents the number of missing values due to technical issues at each time point. *p < 0.05 and ^***^p < 0.001 vs. T1.

Neutrophil phagocytosis of *E. coli* and *S. aureus* bioparticles across the different time points is shown in Figure 2. Phagocytosis capacity for both types of microparticles was not altered directly after the first HBOT session (T2). *S. aureus* bioparticle phagocytosis was significantly decreased before and after the third session (T3 and T4, Figure 2B), whereas a reduction in *E. coli* bioparticle phagocytosis was only observed following the third session (T4, Figure 2A).

### Plasma Cytokine Concentrations

**FIGURE 3:**
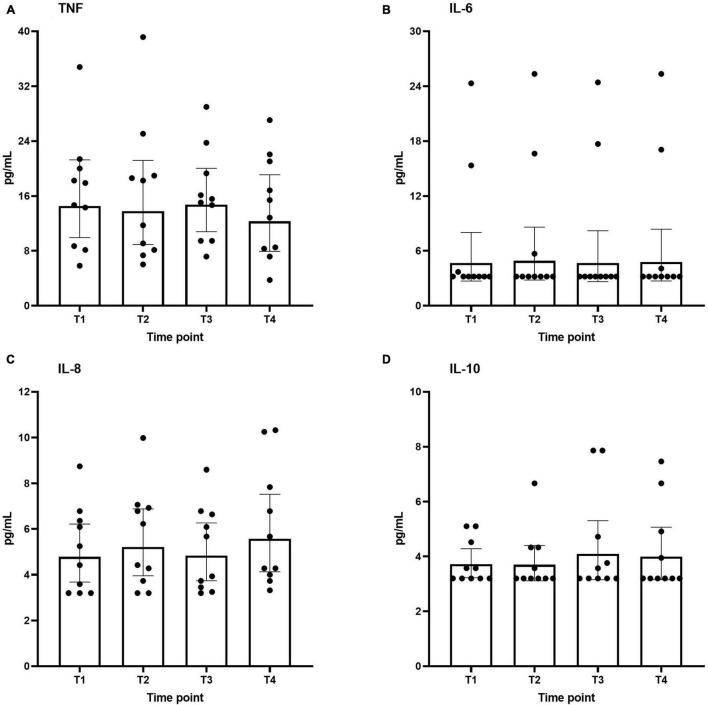
Plasma levels of interleukin IL-6 **(A)**, IL-8 **(B)**, IL-10 **(C)**, and TNF **(D)** during the study period. Data are represented as scatter plots with bars indicating geometric mean and whiskers representing 95% confidence interval (CI).

Plasma concentrations of both pro-inflammatory (TNF, IL-6, and IL-8) and anti-inflammatory (IL-10) cytokines across the different time points are shown in Figure 3. As to be expected in healthy volunteers, circulating cytokine concentrations were very low. Furthermore, no differences were found over time.

### *Ex vivo* Stimulated Cytokine Production

**FIGURE 4:**
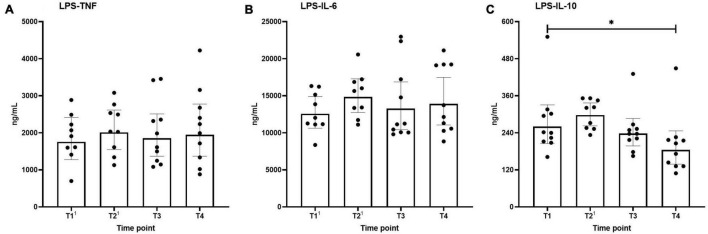
Production of TNF **(A)**, IL-6 **(B)**, and IL-10 **(C)** following *ex vivo* stimulation of leukocytes with LPS. Data are represented as scatter plots with bars indicating geometric mean and whiskers representing 95% confidence interval (CI). Superscript font represents the number of missing values due to technical issues at each time point. *p < 0.05 vs. T1.

**FIGURE 5:**
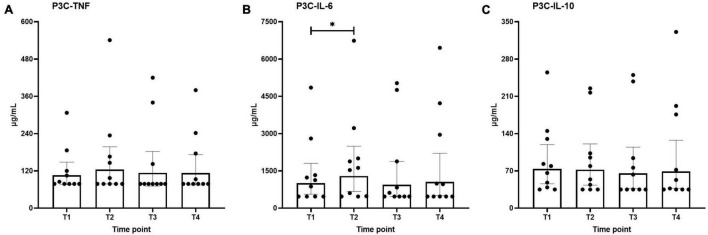
Production of TNF **(A)**, IL-6 **(B)**, and IL-10 **(C)** following *ex vivo* stimulation of leukocytes with Pam3Cys (P3C). Data are represented as scatter plots with bars indicating geometric mean and whiskers representing 95% confidence interval (CI). *p < 0.05 vs. T1.

*Ex vivo*-stimulated production of TNF, IL-6, and IL-10 in response to stimulation with the TLR4 ligand LPS and the TLR2 ligand Pam3Cys across the different time points are shown in Figures 4, 5, respectively. Production of TNF induced by LPS or Pam3Cys showed no differences over time (Figures 4A, 5A). LPS-induced IL-6 production was unaltered at any of the time points (Figure 4B), whereas Pam3Cys-induced IL-6 production was slightly but significantly increased only after the first HBOT-session (Figure 5B, T2). LPS-induced IL-10 production was slightly decreased following the third HBOT session (T4), but no changes were observed at any of the other time points (Figure 4C), and Pam3Cys-induced IL-10 production did not change over time (Figure 5C).

## Discussion

This study is one of the first to investigate the effects of HBOT on oxidative stress and inflammatory parameters in healthy young volunteers. We demonstrate significant decreases in neutrophil intracellular ROS-production and neutrophil phagocytosis after repeated HBOT sessions. HBOT did not induce systemic oxidative stress or increased plasma cytokine levels. Furthermore, the capacity of leukocytes to produce cytokines upon *ex vivo* stimulation was largely unaltered as well.

### Neutrophilic Reactive Oxygen Species Generation and Oxidative Stress

We measured MDA and neutrophil ROS production as parameters of oxidative stress and observed a significant decrease in ROS production by neutrophils after two and three HBOT-sessions. Furthermore, there was a significant reduction in maximum neutrophilic ROS generation capacity (determined by stimulation with PMA) after two HBOT-sessions. Although these results correspond with Morabito et al. (2011), few other studies in healthy volunteers reported an increase in ROS levels during and shortly after HBOT (Labrouche et al., 1999; Ferrer et al., 2007; Bosco et al., 2021). Also, our study showed no alterations in MDA concentrations at any timepoint. Therefore, three regular HBOT-sessions do not result in a degree of oxidative stress that causes significant lipid peroxidation. These findings align with the outcomes of previous studies (Muth et al., 2004; Sureda et al., 2009, 2014; Matzi et al., 2015; Bosco et al., 2018) and the harmless effect of 20–40 sessions commonly used in HBOT protocols.

### Phagocytosis

HBOT may have antimicrobial effects (Memar et al., 2019; Woo et al., 2020). To investigate this, we performed neutrophil phagocytosis. There were no differences in phagocytic activity induced by *E. coli* or *S. aureus* immediately after the first HBOT-session. Yet, we found a decrease in phagocytosis of *S. aureus* before the third HBOT-session and reduced phagocytosis of *S. aureus* and *E. coli* after the third HBOT-session. Our results are in part consistent with a previous study showing that *E. coli* phagocytosis in healthy volunteers is unaltered after single or multiple HBOT-sessions (Labrouche et al., 1999). In contrast, other work reported a significant increase of *S. aureus* phagocytosis during and after exposure to one HBOT-session in healthy volunteers (Jüttner et al., 2003). We hypothesize that the reduced phagocytosis activity may be caused by exhaustion of the neutrophils after multiple HBOT-sessions. Besides, the antimicrobial effect of HBOT, found in previous studies in patients with various conditions, may be absent in healthy volunteers, possibly due to the lack of ongoing inflammation in this group.

### Circulating Cytokine Levels and Cytokine Production Capacity of *ex vivo*-Stimulated Leukocytes

Previous studies reported that the increase in ROS due to HBOT might interfere with cytokine release (Al-Waili and Butler, 2006; Fosen and Thom, 2014). To explore this, we measured plasma concentrations of IL-6, IL-8, IL-10, and TNF as well as of IL-6, IL-10, and TNF production by leukocytes *ex vivo* stimulated with the TLR2 agonist Pam3Cys and the TLR4 agonist LPS. No increases in levels of any of the circulation cytokines were observed. Although a slight increase in Pam3Cys-induced IL-6 production after the first HBOT-session and a slight decrease in LPS-induced IL-10 release following the third session was noted, the effect size was very small and not observed at other time points, questioning its relevance. Our results pertaining to plasma cytokine concentrations correspond to previous work showing differences in circulating TNF levels in healthy volunteers only during HBOT but not afterward and showing no differences in IL-6 plasma concentrations during the entire study period (Rocco et al., 2001). Furthermore, no differences in circulating IL-6, IL-10, or TNF directly after HBOT were reported by others as well (Bosco et al., 2021). However, a study performed in obese individuals reported elevated circulating IL-6 concentrations during and directly after one and four HBOT-session(s) and an increase in TNF levels (Wilkinson et al., 2015). Since obesity is associated with increase IL-6 levels (Wilkinson et al., 2015), this may explain why those results differ from ours. Of note, the same study (Wilkinson et al., 2015), also evaluated mRNA expression of various cytokines in adipose tissue, but no effects of HBOT on these parameters were observed (Wilkinson et al., 2015).

### Limitations

Some limitations to this study may explain why some of our results differ from those reported in previous investigations. First, few studies that investigated the parameters assessed in the present study in healthy volunteers have yielded quite contradictory results, possibly caused by a wide variety in study protocols. Second, to prevent heterogeneity in our relatively small study group, we only included male volunteers aged 18–40 years, which may mean that our results cannot readily be extrapolated to older individuals or females. Third, we only obtained samples before and after the HBOT-sessions and not during the sessions. Samples were decompressed to normal ambient pressure before analysis. Therefore, it was neither possible to determine short-term effects that may only be evident during HBOT and disappear before the analyses were performed, nor are we able to exclude any effect of reducing the ambient pressure before analysis. Fourth, due to the small sample size, partially due to unexpected drop-out of one subject and the laborious determination processes of neutrophil intracellular ROS production and phagocytosis resulting in some missing datapoints due to technical issues, the statistical power is relatively small.

## Conclusion

Three regular HBOT-sessions in healthy volunteers do not induce systemic oxidative stress or a systemic inflammatory response in healthy volunteers. However, our results may suggest that HBOT leads to exhaustion of ROS generation capacity and phagocytosis. These findings provide additional insight into the pathways behind HBOT-induced immunomodulation, but more research into the immunologic effects of HBOT which may underlie the improved clinical outcomes after hyperbaric oxygen preconditioning is necessary.

## Data Availability Statement

The raw data supporting the conclusions of this article will be made available by the authors, without undue reservation.

## Ethics Statement

The studies involving human participants were reviewed and approved by the Medical Research Ethics Committee, Academic Medical Center Amsterdam, Netherlands. The patients/participants provided their written informed consent to participate in this study.

## Author Contributions

RAH, SJ, and MK: study conception and design. SJ: laboratory and data acquisition. SW, RH, SJ, RAH, RW, and MK: data analysis and interpretation. SW and RH: drafting of manuscript. All authors: critical revision of the manuscript for important intellectual content.

## Conflict of Interest

The authors declare that the research was conducted in the absence of any commercial or financial relationships that could be construed as a potential conflict of interest.

## Publisher’s Note

All claims expressed in this article are solely those of the authors and do not necessarily represent those of their affiliated organizations, or those of the publisher, the editors and the reviewers. Any product that may be evaluated in this article, or claim that may be made by its manufacturer, is not guaranteed or endorsed by the publisher.
